# miRNA-302s may act as oncogenes in human testicular germ cell tumours

**DOI:** 10.1038/s41598-019-45573-6

**Published:** 2019-06-24

**Authors:** Mrinal K. Das, Herman S. F. Evensen, Kari Furu, Trine B. Haugen

**Affiliations:** 1 Faculty of Health Sciences, OsloMet – Oslo Metropolitan University, Oslo, Norway; 2 Cancer Registry, Oslo, Norway

**Keywords:** Reverse transcription polymerase chain reaction, Testicular cancer, Cell growth, Cell invasion, miRNAs

## Abstract

Testicular germ cell tumour (TGCT) represents the most common malignancy in young men in large parts of the world, but the aetiology is yet unclear. Multiple TGCT susceptibility loci have been identified, and we have shown that one of these, *SPRY4*, may act as a TGCT oncogene. Furthermore, many of the loci are in non-coding regions of the genome. miRNAs, a class of non-coding RNAs may play a crucial role in cell proliferation, differentiation, and apoptosis, and alteration in their expression may lead to oncogenesis. Differential expression of miRNAs in TGCT and normal testis has been reported in previous studies. In this study, we used qPCR to analyse, in normal and malignant testis tissue, the expression of the ten miRNAs that we had previously identified by sequencing to be the most upregulated in TGCT. We found high expression of these miRNAs also by qPCR analysis. The levels of *miR-302a-3p*, *miR-302b-3p*, and *miR-302c-3p* were downregulated after treatment of the TGCT cell lines NT2-D1 and 833 K with the chemotherapy drug cisplatin. By using miRNA inhibitor-mediated transient transfection, we inhibited the expression of the three members of miR-302 family (miR-302s). Inhibition of miR-302s resulted in a decreased cell proliferation in NT2-D1 cells, but not in 833 K cells. In both cell lines, inhibition of miR-302s resulted in decreased expression of SPRY4, which we have previously shown to regulate MAPK/ERK and PI3K/Akt signalling pathways in these cells. Inhibition of miR-302b-3p and miR-302c-3p decreased phosphorylation of ERK1/2, whereas inhibition of miR-302a-3p and miR-302b-3p led to decreased expression of the apoptosis inhibitor, survivin. Our findings suggest that miR-302s act as TGCT oncogenes by inducing the expression of SPRY4 and activating MAPK/ERK pathway while inhibiting apoptosis via increased survivin expression.

## Introduction

Testicular germ cell tumour (TGCT) represents the most common malignancy in young men in large part of the world, and the incidence is rising^1,2^. The mortality of TGCT has, however, declined over the last decades, mainly due to the introduction of cisplatin-based chemotherapy, and the survival rate is approximately 95% in the Western world^3^. The aetiology of TGCT is yet unclear, and both genetic and environmental factors are believed to contribute to the disease risk^4,5^. Genome-wide association studies (GWAS) have identified more than 50 TGCT susceptibility loci^6,7^, and *SPRY4* is one of the risk genes with strong and consistent association^7–10^. Our results in a previous study, indicate that SPRY4 acts as a TGCT oncogene^11^. Many of the susceptibility loci identified by GWAS are in the non-coding regions of the genome suggesting that non-coding RNAs also influence the development of TGCT. Non-coding RNAs (ncRNAs) may also play a role in TGCT progression^12^.

MicroRNAs (miRNAs), a class of small non-coding RNAs (sncRNAs), play crucial roles in many physiological processes including proliferation, differentiation, and apoptosis, and alterations in expression of miRNAs have been associated with tumourigenesis^13–16^. In a recent study, we showed that miRNAs were one of the most common groups of sncRNAs in TGCT^17^. We also found a different miRNA expression pattern in malignant and normal testis tissue. The largest difference was among members of two clusters, *miR-302/367* and *miR-371-373*, consisting of nine and four miRNAs, respectively. Other studies have also shown high expression of the *miR-302/367* and *miR-371-373* clusters in TGCT tissue as well as in serum samples from TGCT patients^18–21^. High expression of these miRNA clusters in TGCTs indicates that they may act as oncogenes. In a genetic screen-based study, *miR-372* and *miR-373* were shown to act as TGCT oncogenes through inhibition of a tumour suppressor gene, *LATS2*^22^. The role of the *miR-302/367* cluster in TGCTs is yet unknown, however, this cluster has been reported to act as tumour suppressor genes in several other cancers^23–26^.

The primary aim of the current study was to investigate the functional role of selected miRNAs in TGCT development by use of two metastatic TGCT (embryonal carcinoma) cell lines 833 K^27^ and NT2-D1^28^. In our previous study, we analysed the expression pattern of miRNAs mainly by sequencing^17^. In the present study, by using a different approach, i.e. quantitative PCR (qPCR) analysis, we measured the levels of the ten most differentially expressed miRNAs identified in the previous study. We also investigated the effect of the cytotoxic drug cisplatin on the expression of these miRNAs. Subsequently, we inhibited the expression of the most cisplatin-sensitive miRNAs and studied the effect on cell growth, cell death, and cell signalling. We found that miR302s, like SPRY4, were highly expressed in TGCTs and also acted as oncogenes in the TGCT cell lines^11^. We further investigated if there was an association between miR302s and SPRY4 by studying the effect of inhibition of the most cisplatin-sensitive miR302s on SPRY4 expression.

## Methods

### Human tissue samples

For miRNA expression analysis, the TGCT subtypes embryonal carcinoma, seminoma, and mixed germ cell tumour, were bought from Origene (MD, USA), whereas normal adult testis samples were collected from adult organ transplant donors. According to the manufacturer, the mixed germ cell tumour was composed of a mixture of yolk sac tumour, immature teratoma, and mature teratoma. No definite embryonal carcinoma was seen.

The study has been approved by the Regional Committee for Medical and Health Research Ethics – South East Norway (2016/2006, REC South East), and all experiments were performed in accordance with approved guidelines and regulations. For the normal testis samples in connection with organ transplantation, informed consent was obtained according to the Norwegian legislation relating to transplantation, hospital autopsies and the donation of bodies.

### Cell culture

Two TGCT cell lines NT2-D1 and 833 K representing the embryonal carcinoma (EC) were kindly provided by Dr Birgitte Lindeman (Norwegian Institute of Public Health, Oslo). NT2-D1 and 833 K were cultured in DMEM (ATCC, VA, USA) and RPMI-1640 medium (Thermo Fisher Scientific, Massachusetts, USA), respectively, supplemented with 10% foetal bovine serum (Thermo Fisher Scientific, Massachusetts, USA) and 1% Pen/Strep (Thermo Fisher Scientific, Massachusetts, USA) at 37 °C in a humidified 5% CO_2_ incubator. The morphology of both cell lines was regularly investigated, and for use in experiments, stocks of cell lines were passaged no more than ten times.

### miRNA inhibition

miRNA inhibition was performed by following the manufacturer’s instruction (Ambion, CA, USA). Cells were seeded out in a six-well plate and grown overnight. Lipofectamine RNAiMAX (Invitrogen, CA, USA) transfection mix was prepared, and specific miRNA inhibitors (Supplementary Table S1) were used. After 48 hours of transfection, cells were harvested and stored at −70 °C until further use. Inhibition was verified using qPCR analysis.

### Quantitative PCR

Total RNA from cell lines and tissue samples were extracted using RNeasy (Qiagen, CA, USA), and 200 ng of RNA was converted to cDNA using Qiagen miScript II RT Kit (Qiagen, Hilden, Germany). qPCR was performed using 1 ng of cDNA and the Qiagen miScript SYBR Green PCR Kit (Qiagen, Hilden, Germany) under recommended conditions on an AriaMx instrument (Agilent Technologies, Santa Clara, USA). All samples were run in triplicates, and the relative expression was calculated using the equation RQ = 2^−ΔΔCT^. CT values > 35 were regarded as negative. *miR-25-3p* has been shown to be stably expressed in TGCT cells^29^ and was used as a reference gene in our study. The primers used are listed in Supplementary Table S2.

### Western blot

Proteins were isolated after 48-hour transfection using RIPA buffer (Sigma Aldrich, Missouri, USA) containing 150 mM NaCl, 1.0% IGEPAL® CA-630, 0.5% sodium deoxycholate, 0.1% SDS, 50 mM Tris, pH 8.0, phosphatase inhibitors, and protease inhibitors. The total protein concentration was measured using a BCA protein assay kit (Thermofisher Scientific, CA, USA). 30 μg protein was loaded onto 10% Mini-PROTEAN® TGX™ Precast Gels (Bio-Rad Laboratories, CA, USA). After SDS-PAGE, the proteins were blotted onto a PVDF membrane, and the membrane was blocked in TBST with 5% skim milk before incubating with primary antibody overnight at 4 °C. An HRP conjugated secondary antibody was used, and the proteins were detected using the ImageLab machine (Bio-Rad Laboratories, CA, USA).

### Cell survival

Cells (300,000) were seeded out in six-well plate and grown overnight. After 24 hours of incubation with various concentrations of cisplatin (Sigma Aldrich, Missouri, USA), cells were counted using a haemocytometer. The cells were stained with trypan blue before counting to exclude dead cells.

### Cell proliferation/viability

The proliferative capacity of the cells was examined by XTT assay, containing a labelling reagent (XTT) and an electron-coupling reagent (PMS) (Roche, Basel, Switzerland)^30^. The absorbance was measured at 450 nm with a microplate reader after 24 hours of XTT treatment. Cells were incubated with various concentrations of cisplatin for 24 hours and then treated with 50 μl of a mixture of XTT and PMS. Cell proliferation assay was also performed after miRNA inhibition. After 48 hours of transfection with miRNA inhibitors, the cells were seeded out in a 96-well plate and cultured at a density of 3,000 cells/well. At the time points 1 hour, 12 hours, 24 hours, 48 hours, 60 hours, and 72 hours, the cells were treated with 50 μl of XTT solution.

### Cell migration and invasion

For cell migration and invasion assays, the cells were seeded out and grown overnight, followed by transfection with miRNA inhibitors. The transfected cells were grown for 48 hours, followed by serum-deprivation for 24 hours. 50,000 cells were then assayed in each well of a 96-well Boyden Chamber (R&D Systems, Minnesota, USA) for migration and invasion according to the manufacturer’s protocol. 20% foetal bovine serum was used as the chemoattractant. Basement Membrane Extract (R&D Systems, Minnesota, USA) was used as the matrix barrier for the invasion assay.

### Statistical analysis

The results were analysed by *t*-test using the PRISM software. Significant differences were defined by values of p < 0.05.

## Results

### Expression of selected miRNAs in TGCTs

**Figure 1:**
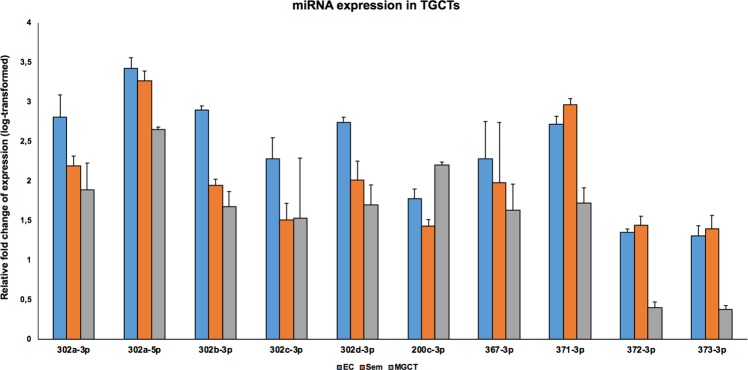
Relative expression levels of ten selected miRNAs in TGCTs. Expression levels of miRNAs in TGCTs were analysed by qPCR. The relative expression levels of the miRNAs were higher in all TGCTs after normalisation to normal testis. Particularly, the expression level of the *miR-302/367* cluster was highest in embryonal carcinoma, the expression of *miR-200c-3p* was highest in mixed germ cell tumours, and the expression of *miR-371-3p* was highest in seminoma. Relative fold change of expression was determined using the equation RQ = 2^−ΔΔCT^; Relative fold change of expression was presented as log-transformed values and the y-axis indicates the exponents. EC: Embryonal carcinoma; Sem: Seminoma; MGT: Mixed germ cell tumours. The designation on the x-axis indicates the miRNA.

We first examined the expression levels of the ten selected miRNAs in normal and malignant testis tissue. These miRNAs were upregulated in all the three TGCT subtypes relative to normal tissue samples (Fig. 1). Notably, the expression of most of the *miR-302/367* cluster members was highest in EC subtype. The expression of these miRNAs was also examined in 833 K and NT2-D1 cells representing EC subtype, in which the members of *miR-302/367* cluster and miR-200c were found to be abundantly expressed, whereas the expression of *miR-371-373* cluster members was not detected (data not shown).

### Effect of cisplatin on miRNAs in TGCT cells

**Figure 2:**
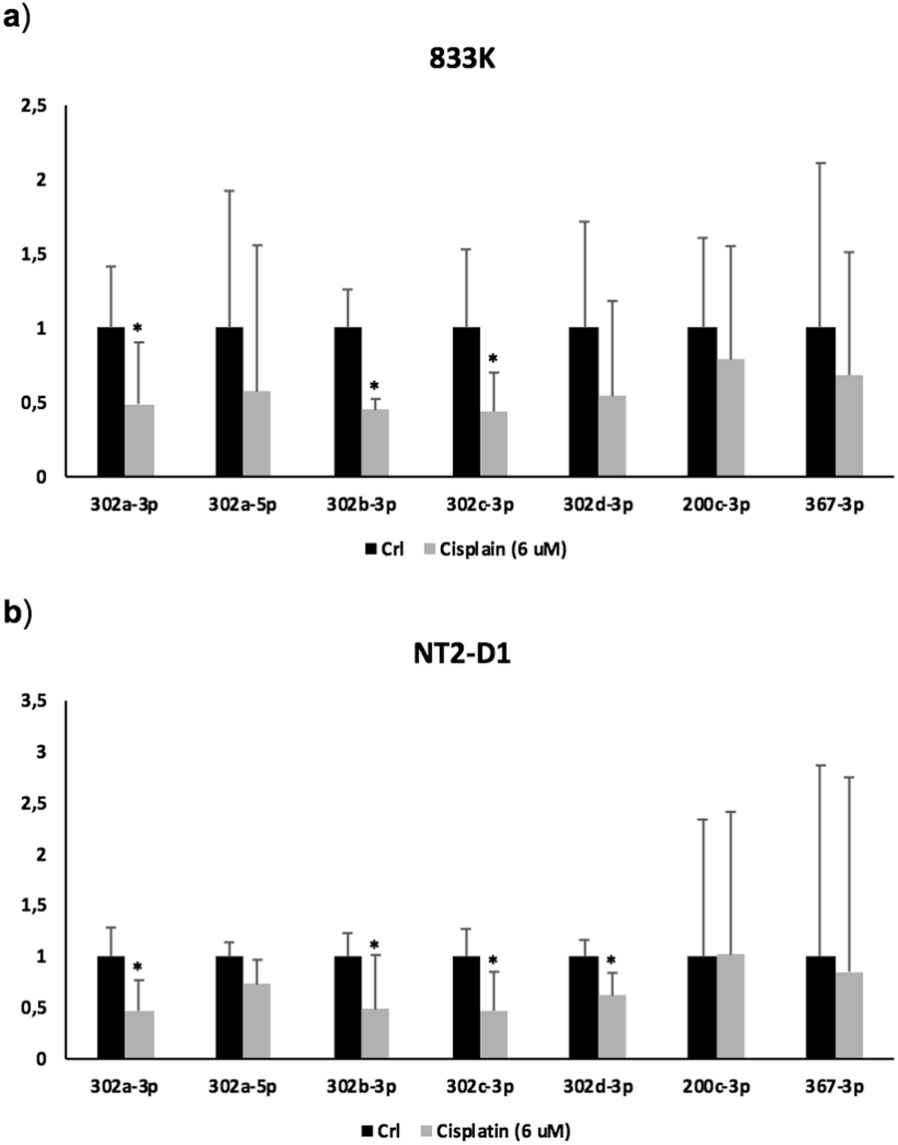
TGCT specific miRNA expression in TGCT cells after cisplatin treatment. The expression of ten miRNAs was analysed by qPCR in both 833 K and NT2-D1 cells after 24-hour incubation with cisplatin (6 μM). Both in 833 K (**a**) and NT2-D1 cells (**b**), the expressions of *miR-302a-3p, miR-302b-3p and miR-302c-3p* were significantly downregulated while the downregulation of *miR-302d-3p* was only significant in NT2-D1 cells. The *miR-302a-5p, miR-200c-3p and miR-367-3p* showed no significant changes, and the expression of the *miR-371-373* cluster was not detected in either cell line. Relative fold change of miRNA expression was determined using the equation RQ = 2^−ΔΔCT^. *t*-test: Control vs Cisplatin (6 μM), mean ± SD (calculated from three independent experiments), statistical significance p < 0.05. The designation on the x-axis indicates the miRNA.

IC_50_ dose (a concentration causing approximately 50% inhibition of the desired activity) of cisplatin was first determined to be 6 μM in 833 K and NT2-D1 cells by assessing cell survival and cell proliferation (Supplementary Fig. S1). Subsequently, the expression of the selected ten miRNAs was analysed by qPCR in both cell lines after 24-hour incubation with cisplatin (Fig. 2). Cisplatin treatment in 833 K and NT2-D1 cells resulted in a significant downregulation of *miR-302a-3p, miR-302b-3p*, and *miR-302c-3p*. The expression of *miR-302d-3p* was only significantly downregulated in NT2-D1 cells, whereas *miR-302a-5p*, *miR-200c*, and *miR-367-3p* showed no significant changes in either of the cell lines.

### Inhibition of miR302s in TGCT cells

**Figure 3:**
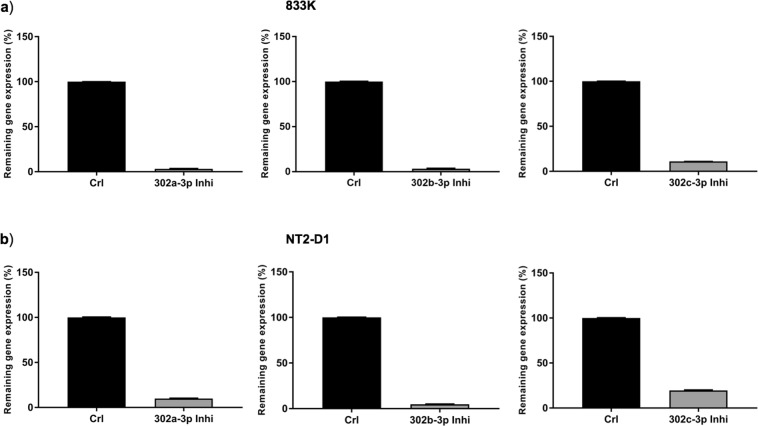
Inhibition of miR302a-3p, miR-302b-3p, and miR-302c-3p in TGCT cells. miRNA inhibitor-mediated transient transfection reduced the expression levels of all three miRNAs by more than 80% in 833 K (**a**) and NT2-D1 cells (**b**), as analysed by qPCR. A non-targeting, miRNA inhibitor negative control with the same chemical modifications was used as a vehicle control. Relative fold change of expression was determined using the equation RQ = 2^−ΔΔCT^. The designation on the x-axis indicates the miRNA inhibitor.

To further study the functional role of the miRNAs shown to be differentially expressed upon cisplatin treatment, we selected miR302a-3p, miR-302b-3p and miR-302c-3p (miR-302s), and performed miRNA inhibitor-mediated transient transfection in 833 K and NT2-D1 cells. The transfection resulted in more than 80% inhibition of the expression for all three miRNAs (Fig. 3).

### Effect of inhibition of the miR-302s on cell proliferation, migration, and invasion

**Figure 4:**
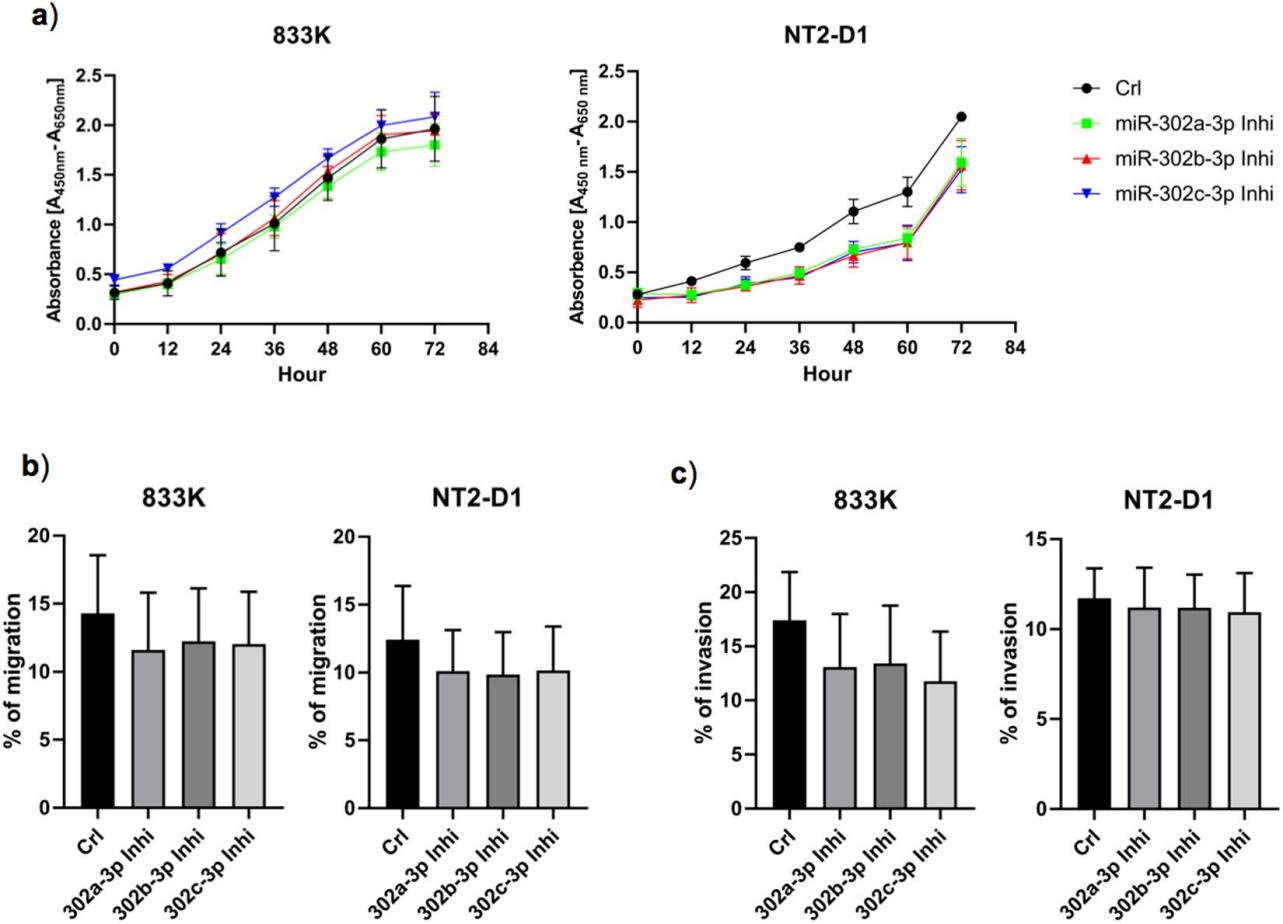
Cell proliferation, migration, and invasion in TGCT cells after inhibition of miR-302s. Inhibition of miR-302s resulted in a significant decrease of cell proliferation in NT2-D1 cells in a time-dependent manner, whereas no significant change was observed in 833 K cells (**a**). Inhibition of miR-302s did not result in a significant decrease in cell migration and invasion in either cell line (**b**,**c**). *t*-test: Control vs miRNA inhibitor, mean ± SD (calculated from three independent experiments), statistical significance p < 0.05. In figure b and c, the designation on the x-axis indicates the miRNA inhibitor.

Inhibition of miR-302s resulted in a significant reduction in cell proliferation in NT2-D1 cells in a time-dependent manner, whereas no significant change was observed in 833 K cells after miRNA inhibition (Fig. 4a). Inhibition of miR-302s in 833 K and NT2-D1 cells did not result in a significant reduction in cell migration and invasion, however, a tendency towards reduction in migration and invasion was observed (Fig. 4b,c).

### Effect of inhibition of the miR-302s on SPRY4, MAPK/ERK signalling, and apoptosis

**Figure 5:**
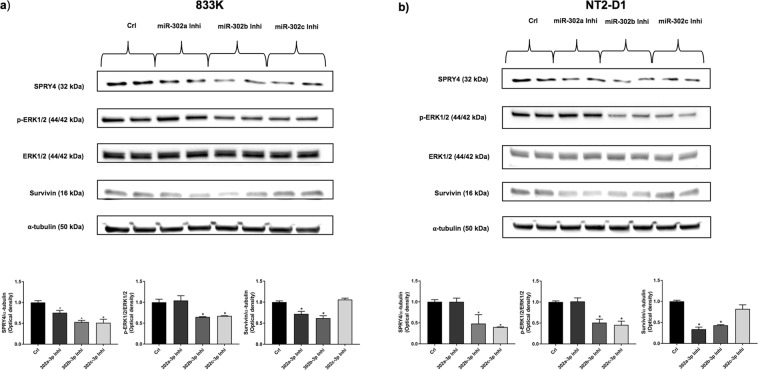
Effect of inhibition of miR-302s on SPRY4, MAPK/ERK signalling and apoptotic inhibitor. Western blot was performed to investigate the expression of SPRY4, phosphorylation of ERK1/2 and the apoptotic inhibitor survivin after inhibition of miR-302s. Inhibition of miR-302s significantly reduced the expression of SPRY4 in both cell lines (**a**,**b**) while inhibition of miR-302b-3p and miR-302c-3p significantly decreased phosphorylation of ERK1/2 in 833 K (**a**) and NT2-D1 cells (**b**). After inhibition of miR-302a-3p and miR-302b-3p, the expression of survivin, an inhibitor of apoptosis, decreased significantly in 833 K (**a**) and NT2-D1 cells (**b**). ERK1/2 was used as endogenous control for phospho-ERK1/2 (p-ERK1/2), and α-tubulin was used as a loading control. The bar graphs show the corresponding densitometric analyses of the western blots where the ratio of p-ERK1/2/ERK1/2 was calculated after normalising to α-tubulin. SPRY4 and survivin were also normalised to α-tubulin. Samples were loaded as duplicates from two independent experiments. The cropped blots are used in the figure, and full-length blots are presented in Supplementary Figs S2 and S3. The experiments were repeated at least three times, and a representative experiment is shown. *t-*test: Control vs miRNA inhibitor, mean ± SD, statistical significance p < 0.05. The designation on the x-axis indicates the miRNA inhibitor.

Suppressing the expression of miR-302s significantly reduced the expression of SPRY4 (Fig. 5a,b). Inhibition of miR-302b-3p and miR-302c-3p significantly decreased the phosphorylation of ERK1/2 in NT2-D1 and 833 K cells, whereas inhibition of miR-302a-3p and miR-302b-3p resulted in a significant decrease in survivin protein expression, an inhibitor of apoptosis (Fig. 5a,b). We also examined the expression level of survivin in the tissue. We found survivin in all the TGCTs except yolk sac tumour, whereas no detection of survivin was observed in normal testis (Supplementary Fig. S4).

## Discussion

Several miRNAs have been reported to be associated with cancer development and are believed to function as either tumour suppressor genes or oncogenes^31^. Moreover, about 50% of annotated human miRNAs were found to be mapped in genomic areas, distinguished as cancer-related chromosomal fragile sites^32^. Though differential expression of miRNAs in TGCTs is well documented, only a few miRNAs have functionally been implicated in TGCT development^17,18,22^.

In the present study, we confirmed with qPCR analysis, that the ten most differentially expressed miRNAs found in our sequencing study were highly expressed in TGCTs compared to the normal testis. Particularly, expression of the *miR-302/367* cluster members was highest in the embryonal carcinoma subtype. High expression of *miR-302s* has not been reported in cancers other than TGCTs. A low or barely detectable expression of *miR-302s* was reported in hepatocellular carcinoma^23^, gastric cancer^33^, colon cancer^34^, and cervical carcinoma^24^. Human embryonic stem cells (hESCs) and induced pluripotent stem cells, however, showed high expression of *miR-302s*, which declined rapidly after differentiation^35^. High expression of *miR-302s* in TGCTs may suggest that they act as oncogenes in TGCTs. After cisplatin treatment in 833 K and NT2-D1 cells, we found that the expressions of *miR-302a-3p*, *miR-302b-3p*, and *miR-302c-3p* were downregulated. It is evident that treatment with cisplatin in cancer cells alters the expression of genes which are functionally involved in the disease mechanism^36^. In our study, downregulation of miR-302s in TGCT cells upon cisplatin treatment as well as the high expression in TGCTs, could indicate that miR-302s play a role in TGCT pathogenesis. However, it is also possible that this result reflects merely a role in cisplatin response.

The effect of miR-302s on cell proliferation may vary depending on cell type. In our study, inhibition of miR-302s resulted in a decreased proliferation of NT2-D1 cells, but not of 833 K cells. A decreased cell proliferation was also observed with hESCs after suppressing the expression of miR-302s^37^. hESCs and human embryonal carcinoma cells (hECCs) have been reported to share overlapping metabolic signatures^38^, similar gene, protein, and miRNA expression profiles^39,40^. Li *et al*. showed that, like hECCs, hESCs expressing high levels of miR-302s could form teratomas *in vivo*, and suppressing the expression of miR-302s in hESCs resulted in reduced teratoma formation^37^. In contrast to the oncogenic properties of miR-302s as indicated in TGCT cells, overexpression of these miRNAs has been shown to suppress cell proliferation in other cancer cells^23–26^. Furthermore, overexpression of miR-302s inhibited cell invasion and migration in melanoma^34^, colon^34^, osteosarcoma^41^, and colorectal cancer cells^42^. Although inhibition of miR-302s in our study did not result in a significant decrease in cell migration and invasion, a tendency towards decline was observed. These differences may reflect a differing mode of action of miRNAs in different cell types, probably through regulating different target genes^43^. The same miRNA may have different roles in various cellular contexts^44^. For example, Chen *et al*. illustrated that ectopic expression of miR-181 had different effects on the differentiation of B cells and cytotoxic T cells^45^. miR-20a and miR-290 showed a pro-senescence role in mouse embryonic fibroblasts^46,47^, whereas in tumours and mouse ESCs, a proliferative role was observed^48,49^.

The miR-302-367 cluster has been demonstrated to target regulatory proteins associated with cell signalling, cell cycle, and cell death^50,51^. We found that inhibition of miR-302s reduced the expression of SPRY4, which is a regulator of MAPK/ERK and PI3K/Akt signalling pathways^52^. In our previous study, we showed that SPRY4 was highly expressed in TGCTs, but no expression was detected in normal testis, and suppression of *SPRY4* in TGCT cells attenuated cell growth, migration, invasion, and phosphorylation of ERK1/2 and Akt^11^. Inhibition of miR-302b-3p and miR-302c-3p also resulted in a decreased phosphorylation of ERK1/2 in both cell lines. To our knowledge, our study is the first demonstrating that suppression of miR-302s inhibits the activation of MAPK/ERK signalling pathway in any cancer type. An opposite finding was reported by Wei *et al*., in which overexpression of miR-302a inhibited ERK1/2 phosphorylation in colorectal cancer cells^42^. They also showed that overexpression of miR-302a decreased Akt phosphorylation. However, we found no significant changes of Akt phosphorylation in 833 K and NT2-D1 cells after inhibition of miR-302s (data not shown). Inhibition of miR-302s in TGCT cells resulting in decreased expression of SPRY4 and reduced phosphorylation of ERK1/2 suggests that miR-302s may act as oncogene in TGCTs by targeting a tumour suppressor gene upstream SPRY4.

In our study, we also showed that inhibition of miR-302a-3p and miR-302b-3 suppressed the expression of survivin in NT2-D1 and 833 K cells. Survivin (encoded by *BIRC5*), an inhibitor of apoptosis, has been widely studied in malignancies, and high expression of survivin is a hallmark of virtually all human tumours including TGCTs^53,54^. We confirmed high expression of survivin in various TGCT samples. Decreased expression of survivin in TGCT cells upon suppression of miR-302s indicates that these miRNAs may inhibit apoptosis in TGCTs, possibly acting through the mechanism of increasing expression of survivin. Divergences in the findings in the two EC cell lines NT2-D1 and 833 K may reflect the heterogeneity of the cell lines. Both cell lines show the characteristics of EC cells, and NT2-D1 also comprises teratoma^28^, whereas 833 K comprises seminoma and choriocarcinoma cells in addition to EC and teratoma^27^. This histologic difference indicates that the 833 K cells may harbour more complex microenvironment than the NT2-D1 cells.

In conclusion, miR-302s may act as TGCT oncogenes via inducing the expression of SPRY4 and activating MAPK/ERK pathway while inhibiting apoptosis via increasing the expression of survivin. More mechanistic studies are needed to understand the role of miR-302s in TGCT pathogenesis.

## Supplementary information

Dataset 1

## Data Availability

The datasets generated during and/or analysed during the current study are available from the corresponding author on reasonable request.
