# Supplementary material for: miRNA-302s may act as oncogenes in human testicular germ cell tumours
Source: Sci Rep. 2019 Jun 24;9:9189. doi: 10.1038/s41598-019-45573-6 (PMC6591358; doi:10.1038/s41598-019-45573-6)
Supplement: Supplementary file 1 — Dataset 1 [file 41598_2019_45573_MOESM1_ESM.docx]

# Supplementary Information

# for

# miRNA-302s may act as oncogenes in human testicular germ cell tumours

Mrinal K. Das^1*^, Herman F. Evensen^1^, Kari Furu^1,2#^ & Trine B. Haugen^1#^

^1^Faculty of Health Sciences, OsloMet – Oslo Metropolitan University, Oslo, Norway

^2^Cancer Registry, Oslo, Norway

*corresponding author, email: [mrinal-kumar.das@oslomet.no](mailto:mrinal-kumar.das@oslomet.no)

#shared the last authorship

Keywords: miR-302, testis, germ cell tumour, miRNA inhibition, cell proliferation

## Supplementary information includes:

1. Supplementary Table S1
2. Supplementary Table S2
3. Supplementary Figure S1
4. Supplementary Figure S2
5. Supplementary Figure S3
6. Supplementary Figure S4

# Supplementary Table S1: List of miRNA inhibitors

| **miRNA ID** | **Name** | **Distributor** | **Sequence** |
| --- | --- | --- | --- |
|  | mirVana™ miRNA Inhibitor, negative control #1 | Ambion, Thermo Fisher Scientific | Not provided |
| hsa-miR-302a-3p | mirVana® miRNA inhibitor | Ambion, Thermo Fisher Scientific | Not provided |
| hsa-miR-302b-3p | mirVana® miRNA inhibitor | Ambion, Thermo Fisher Scientific | Not provided |
| hsa-miR-302c-3p | mirVana® miRNA inhibitor | Ambion, Thermo Fisher Scientific | Not provided |

# Supplementary Table S2: List of miRNA primer assays

| **Gene symbol** | **Assay name** | **Assay ID** | **Distributor** | **Sequence** |
| --- | --- | --- | --- | --- |
| *hsa-miR-302a-3p* | Hs_miR-302a_2 miScript Primer Assay | MS00009331 | Qiagen | 5'UAAGUGCUUCCAUGUUUUGGUGA |
| *hsa-miR-302a-5p* | Hs_miR-302a*_3 miScript Primer Assay | MS00009338 | Qiagen | 5'ACUUAAACGUGGAUGUACUUGCU |
| *hsa-miR-302b-3p* | Hs_miR-302b_1 miScript Primer Assay | MS00003906 | Qiagen | 5'UAAGUGCUUCCAUGUUUUAGUAG |
| *hsa-miR-302c-3p* | Hs_miR-302c_1 miScript Primer Assay | MS00003913 | Qiagen | 5'UAAGUGCUUCCAUGUUUCAGUGG |
| *hsa-miR-302d-3p* | Hs_miR-302d_1 miScript Primer Assay | MS00003920 | Qiagen | 5'UAAGUGCUUCCAUGUUUGAGUGU |
| *hsa-miR-367-3p* | Hs_miR-367_2 miScript Primer Assay | MS00009583 | Qiagen | 5'AAUUGCACUUUAGCAAUGGUGA |
| *hsa-miR-200c-3p* | Hs_miR-200c_1 miScript Primer Assay | MS00003752 | Qiagen | 5'UAAUACUGCCGGGUAAUGAUGGA |
| *hsa-miR-371a-3p* | Hs_miR-371_1 miScript Primer Assay | MS00004060 | Qiagen | 5'AAGUGCCGCCAUCUUUUGAGUGU |
| *hsa-miR-372-3p* | Hs_miR-372_1 miScript Primer Assay | MS00004067 | Qiagen | 5'AAAGUGCUGCGACAUUUGAGCGU |
| *hsa-miR-372-3p* | Hs_miR-373_2 miScript Primer Assay | MS00031815 | Qiagen | 5'GAAGUGCUUCGAUUUUGGGGUGU |
| *hsa-miR-25-3p* | Hs_miR-25_1 miScript Primer Assay | MS00003227 | Qiagen | 5'CAUUGCACUUGUCUCGGUCUGA |

# Supplementary Figure S1

**b)**

**a)**


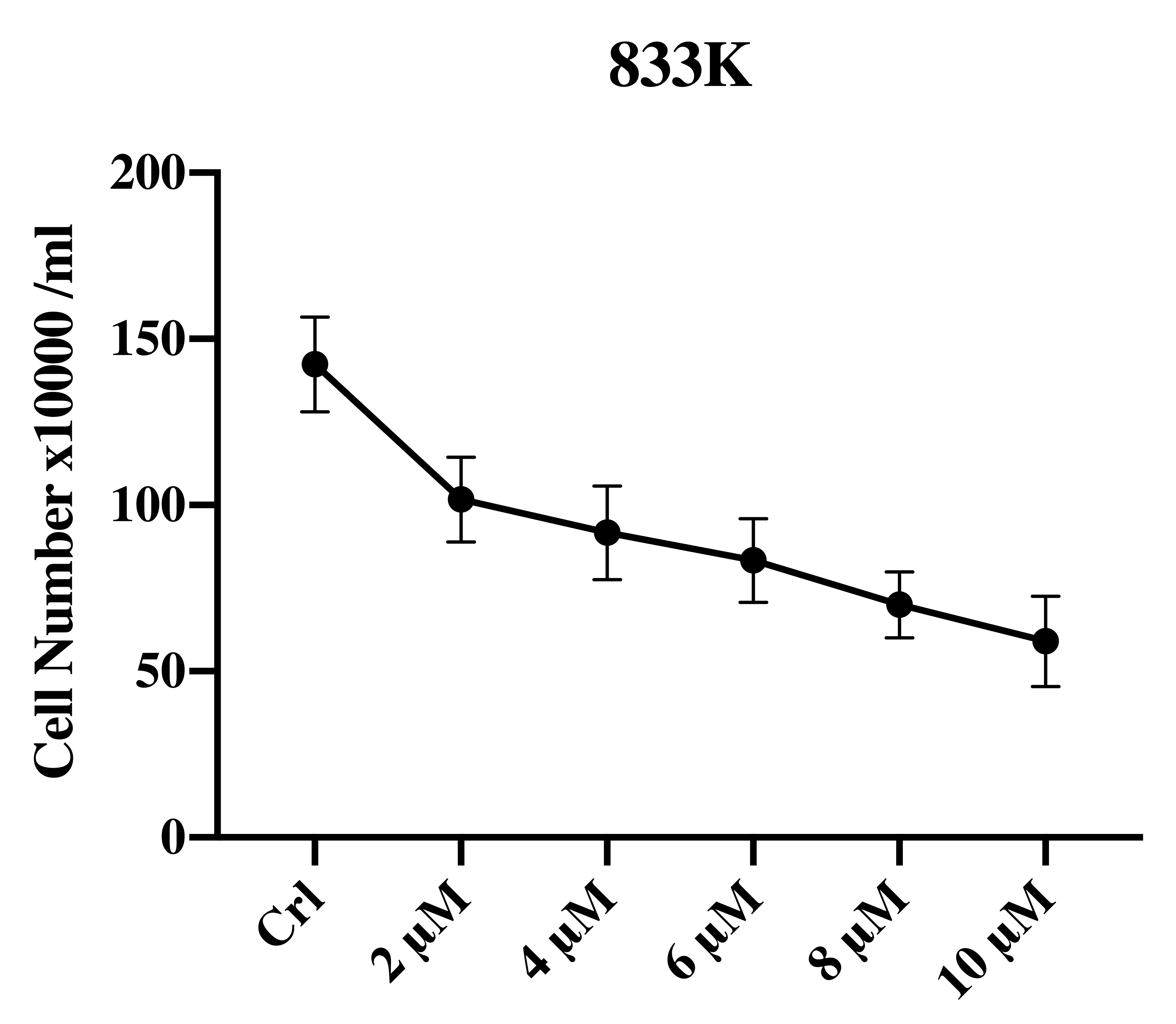

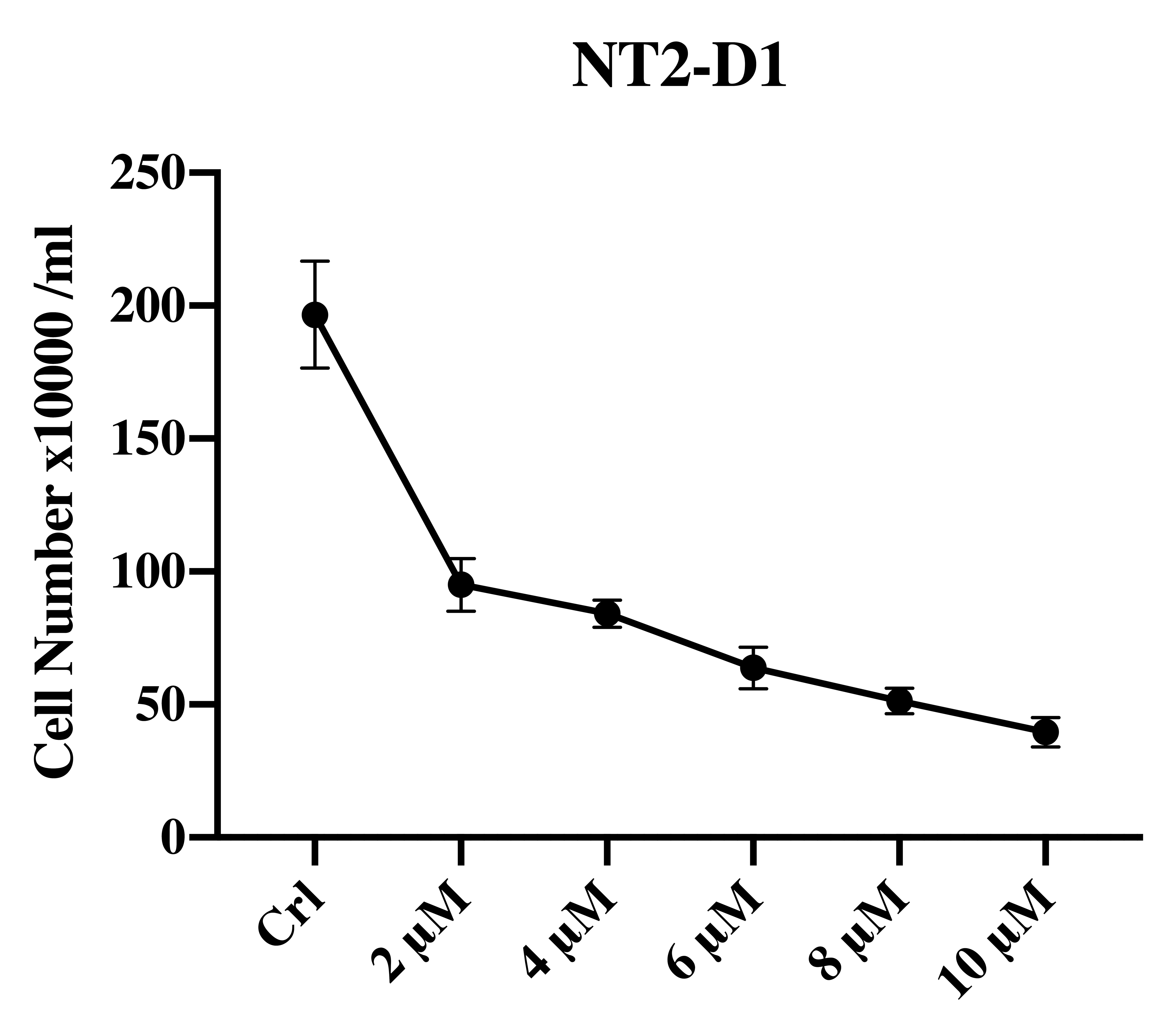

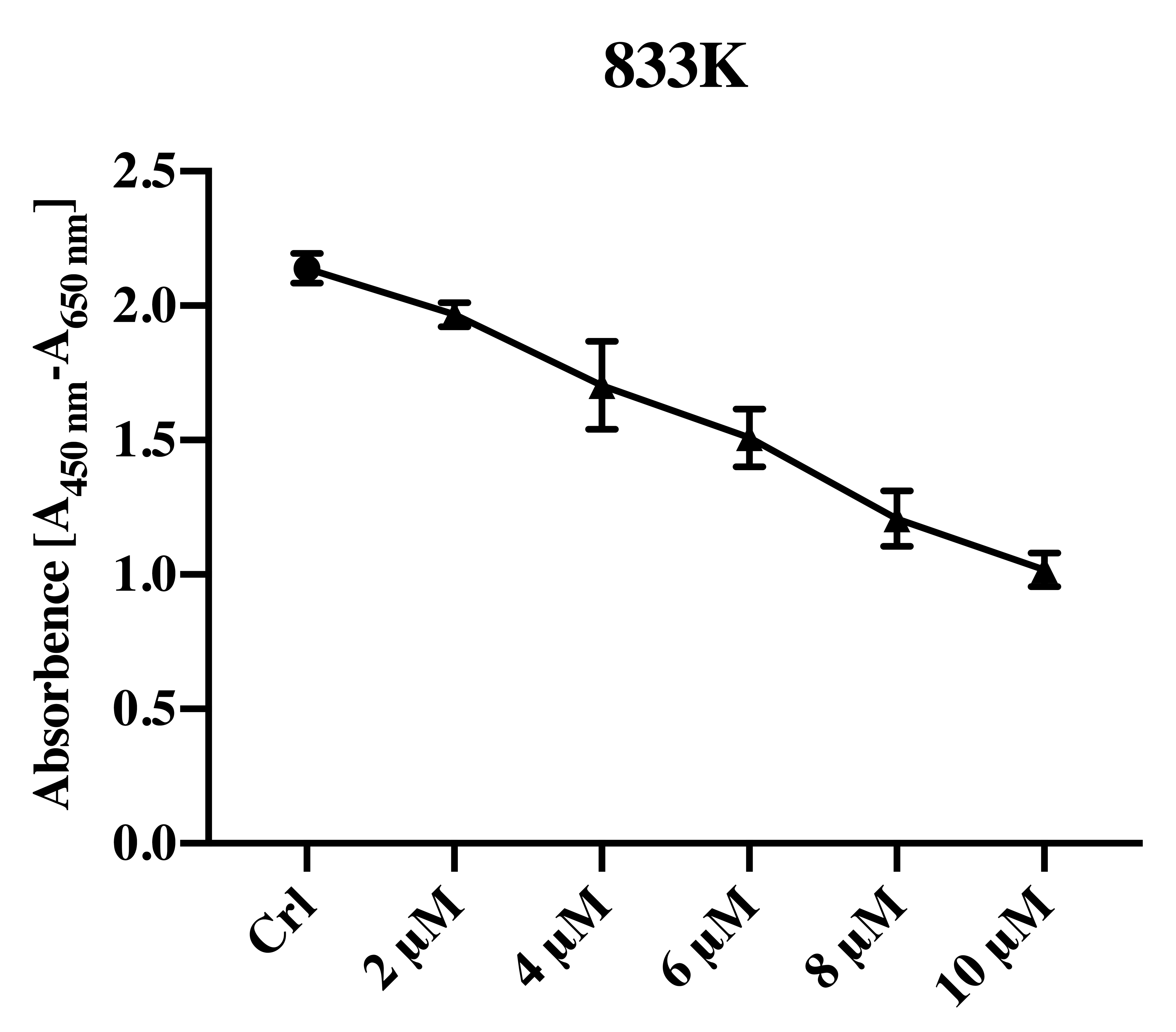

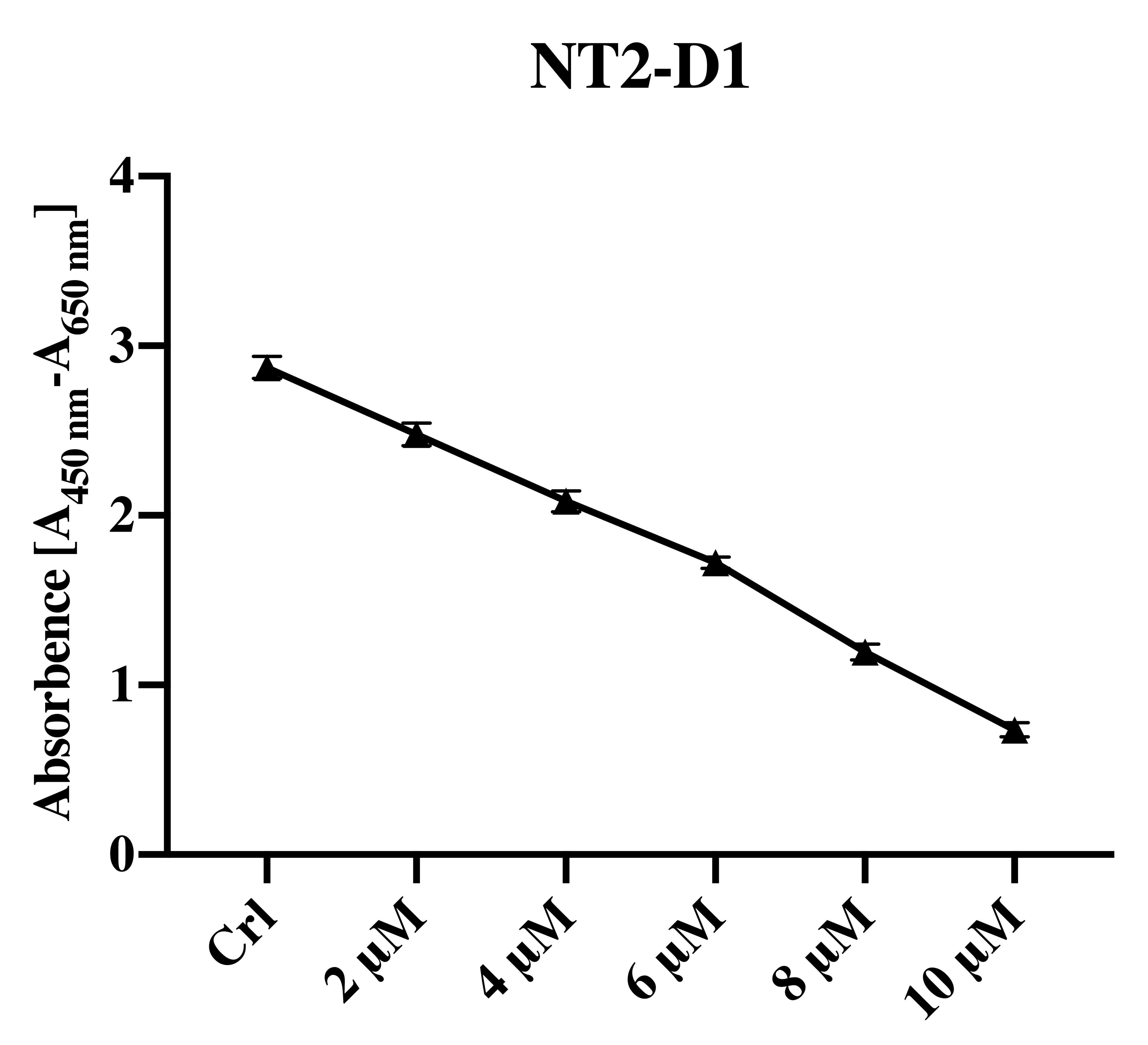


**c)**

**d)**

**Supplementary Figure S1**: Cell cultures were treated with five different concentrations of cisplatin (2 μM to 10 μM) for 24 hours before measuring. Cell viability and proliferation significantly decreased both in 833K (a, c) and NT2-D1 cells (b, d) in a dose-dependent manner. *t*-test: Control vs Cisplatin, mean ± SD (calculated from three independent experiments), statistical significance p < 0.05.

# Supplementary Figure S2


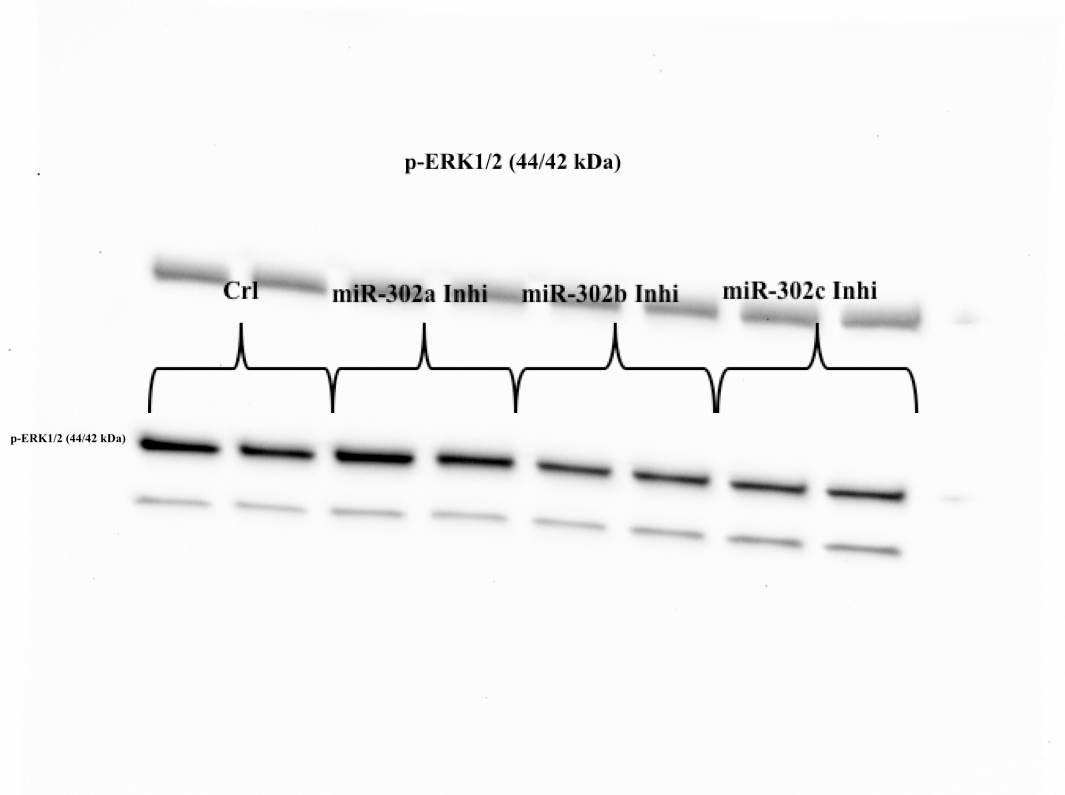

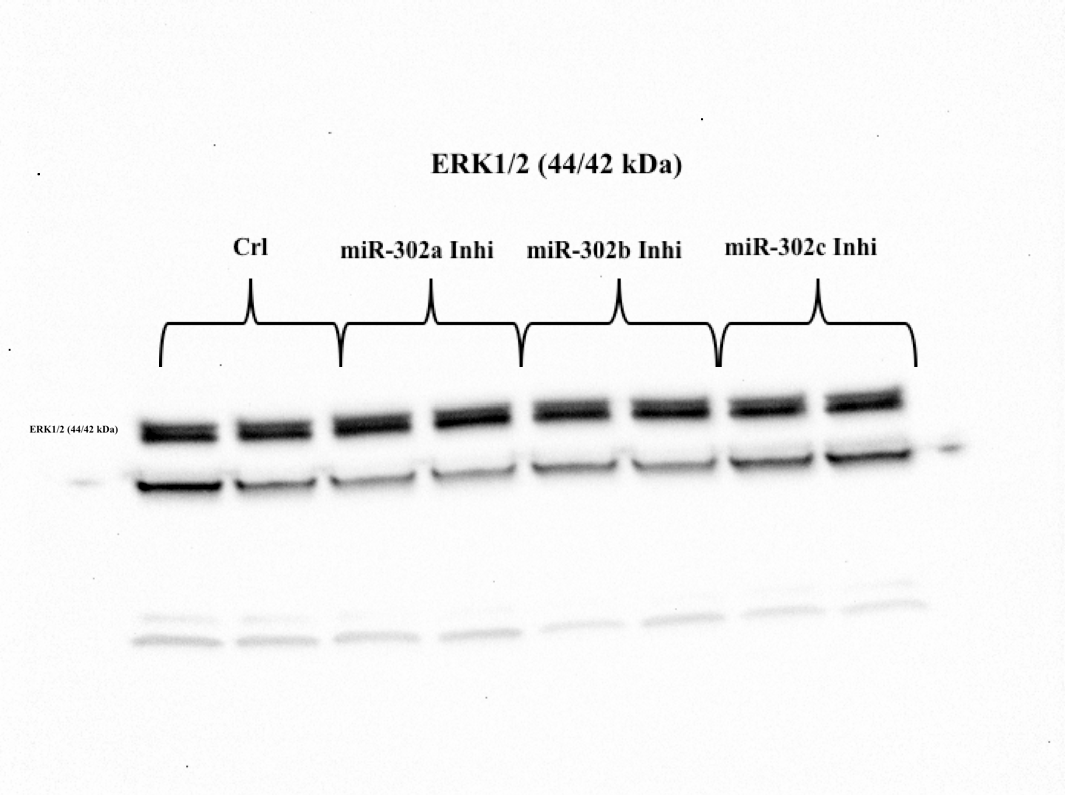


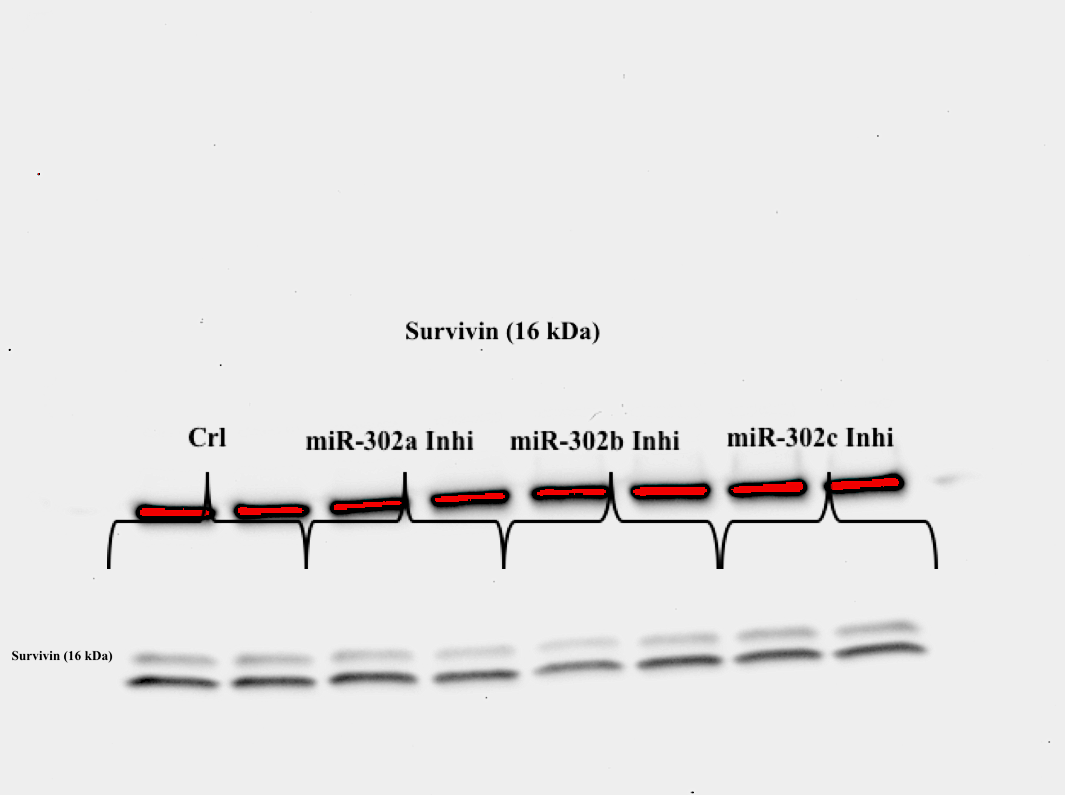

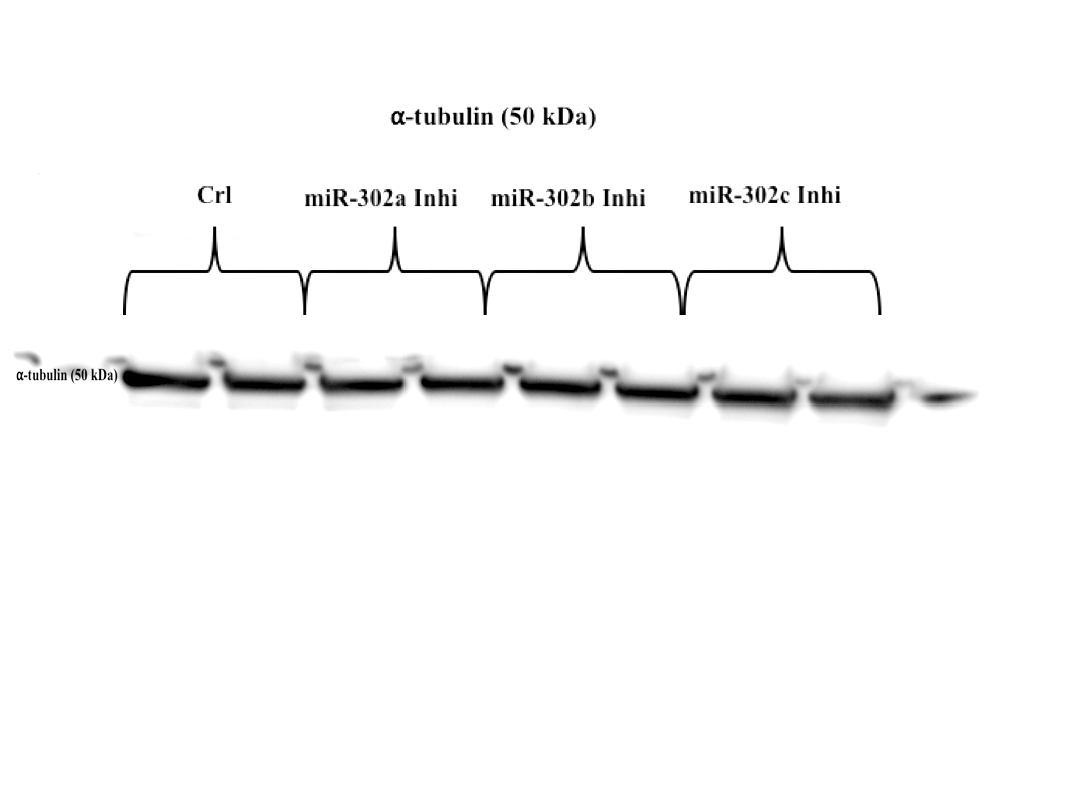


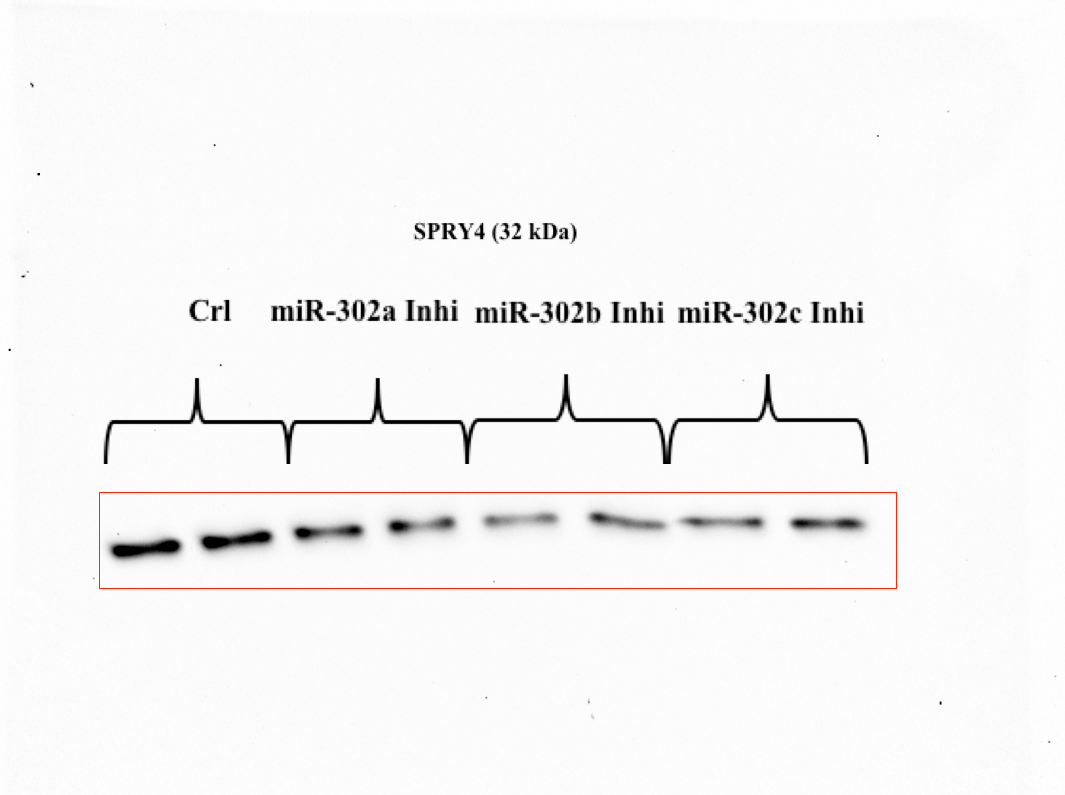

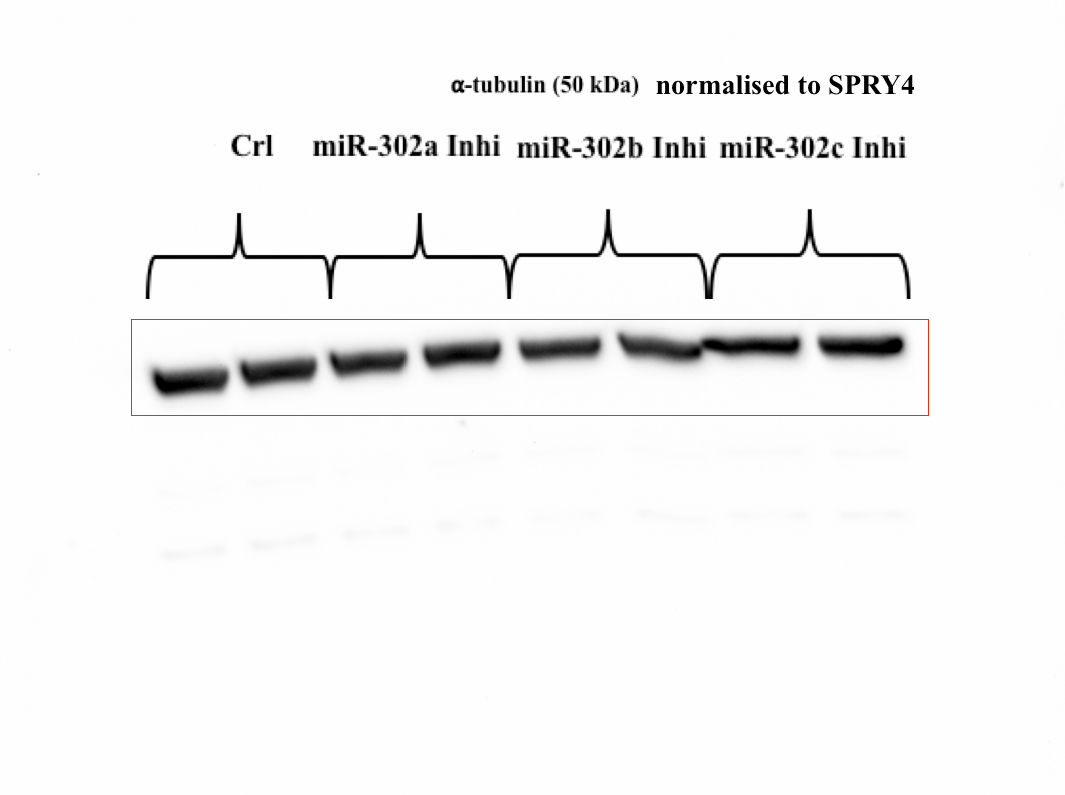


**Supplementary Figure S2**: Full length blots of Fig 5a. Red rectangle shows the cropping location.

# Supplementary Figure S3


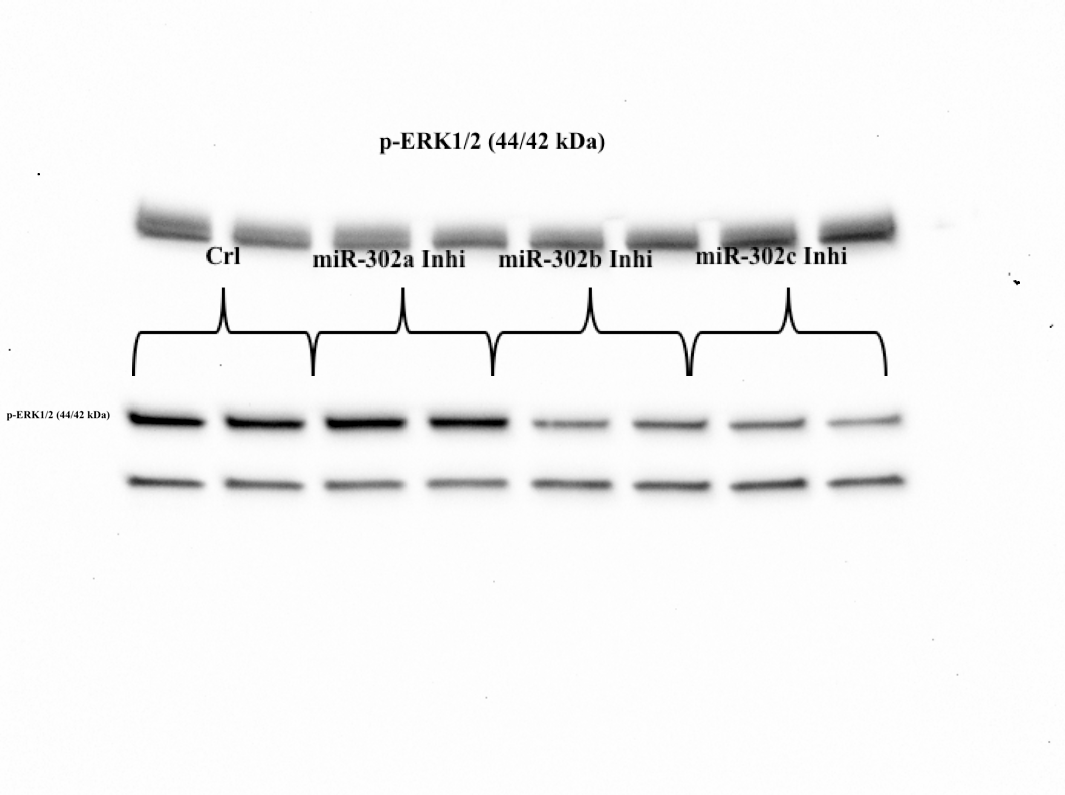

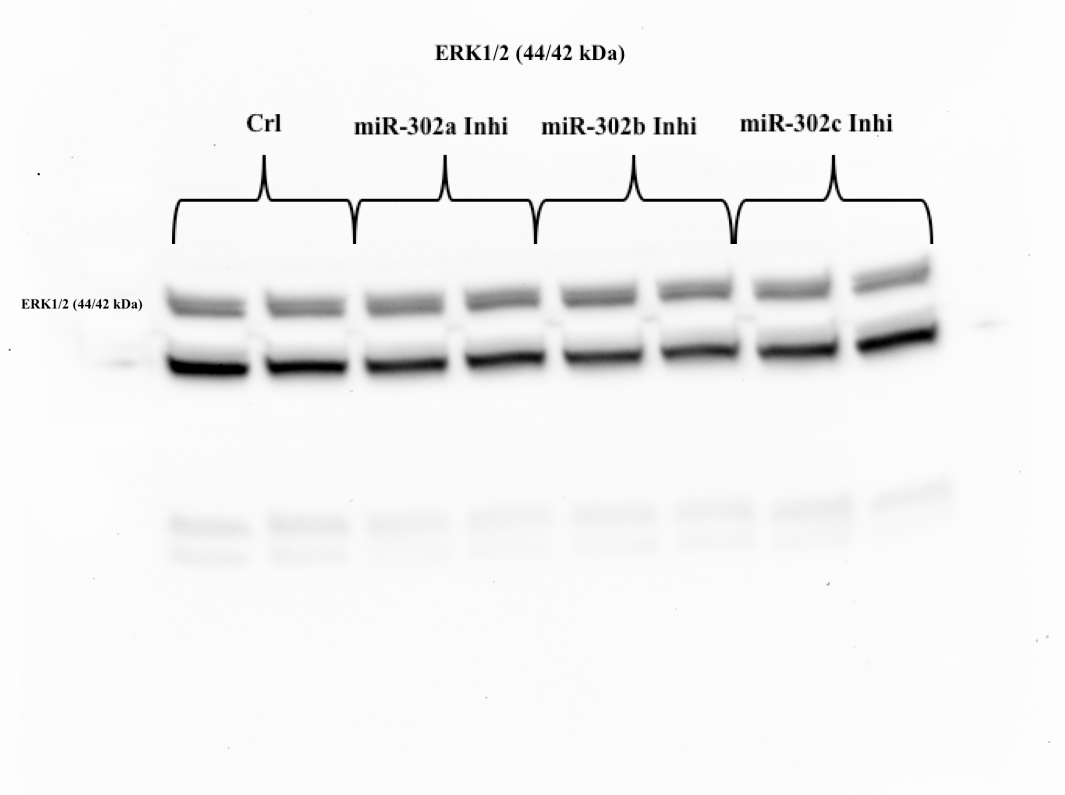


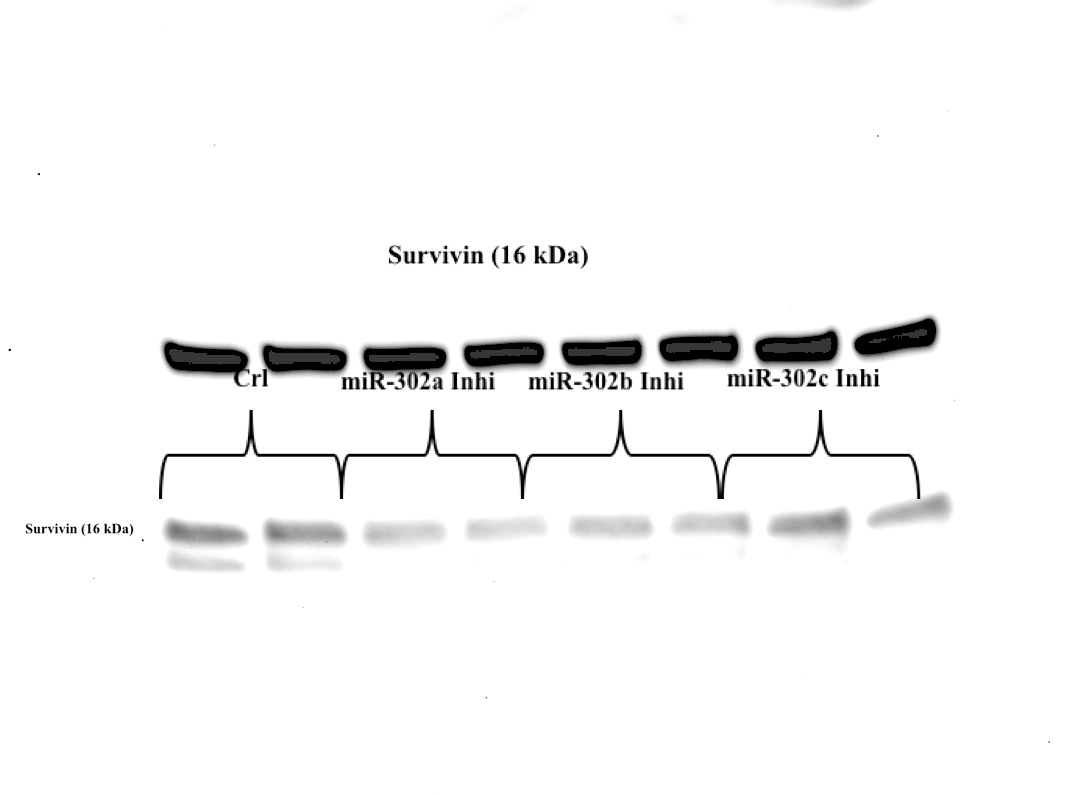

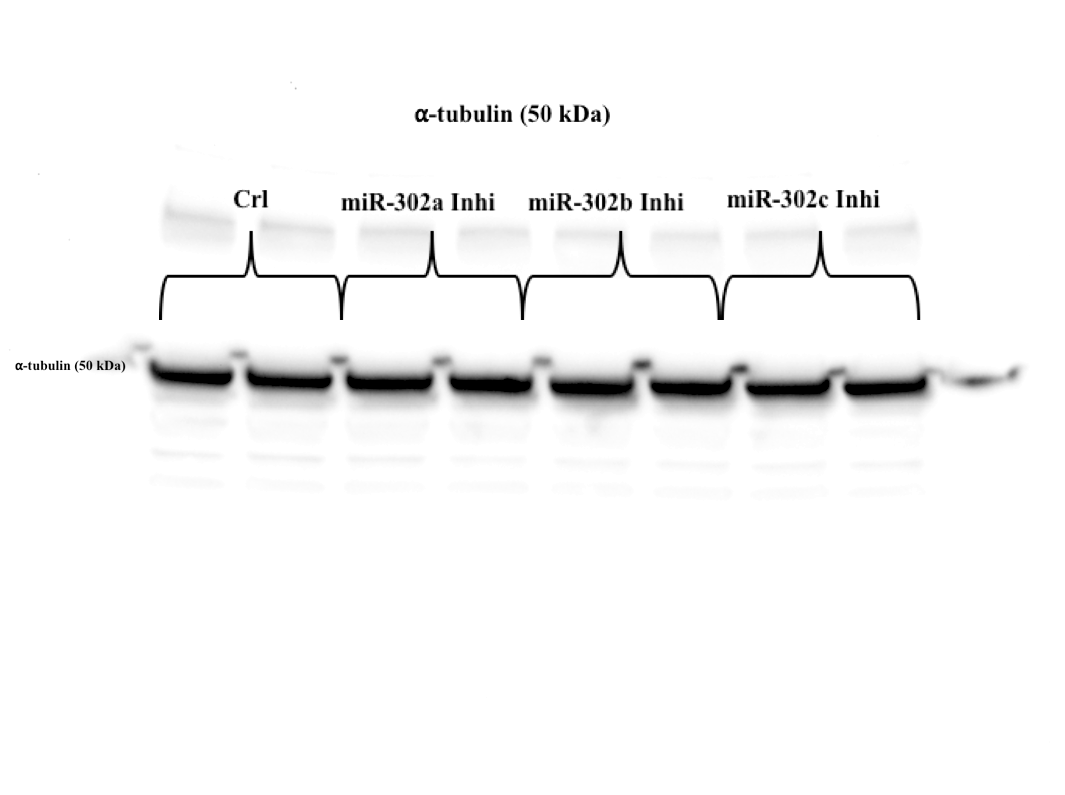


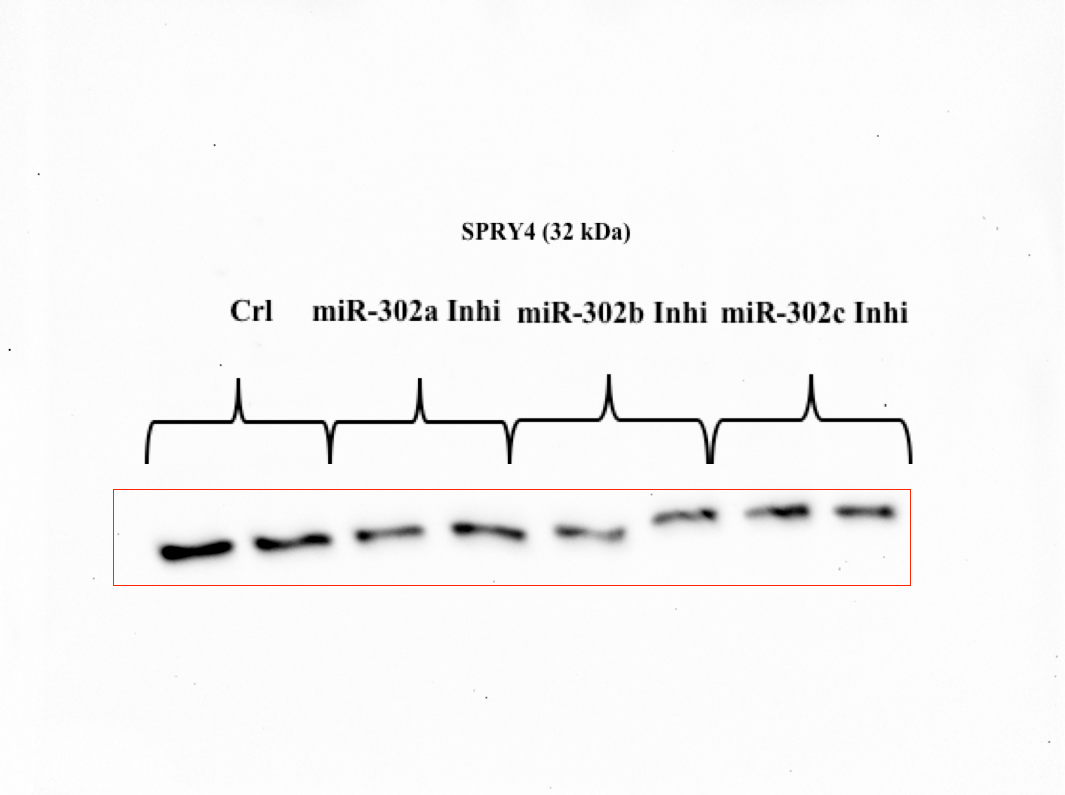

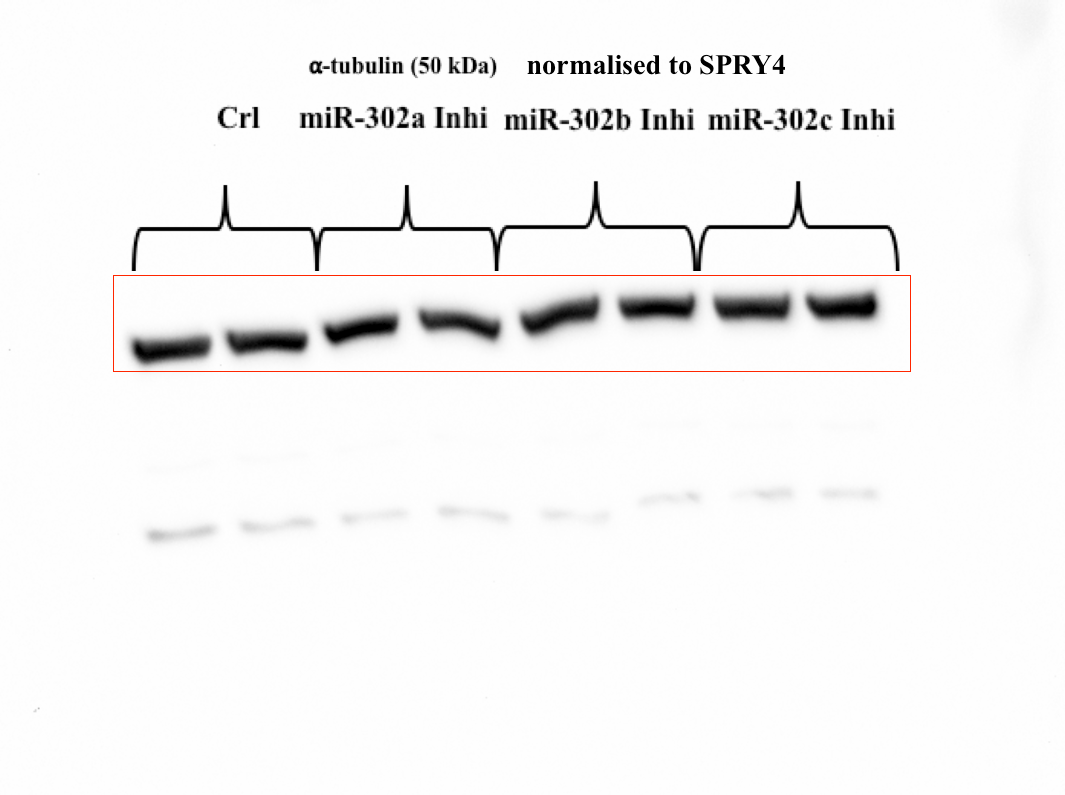


**Supplementary Figure S3**: Full length blots of Fig 5b. Red rectangle shows the cropping location.

# Supplementary Figure S4


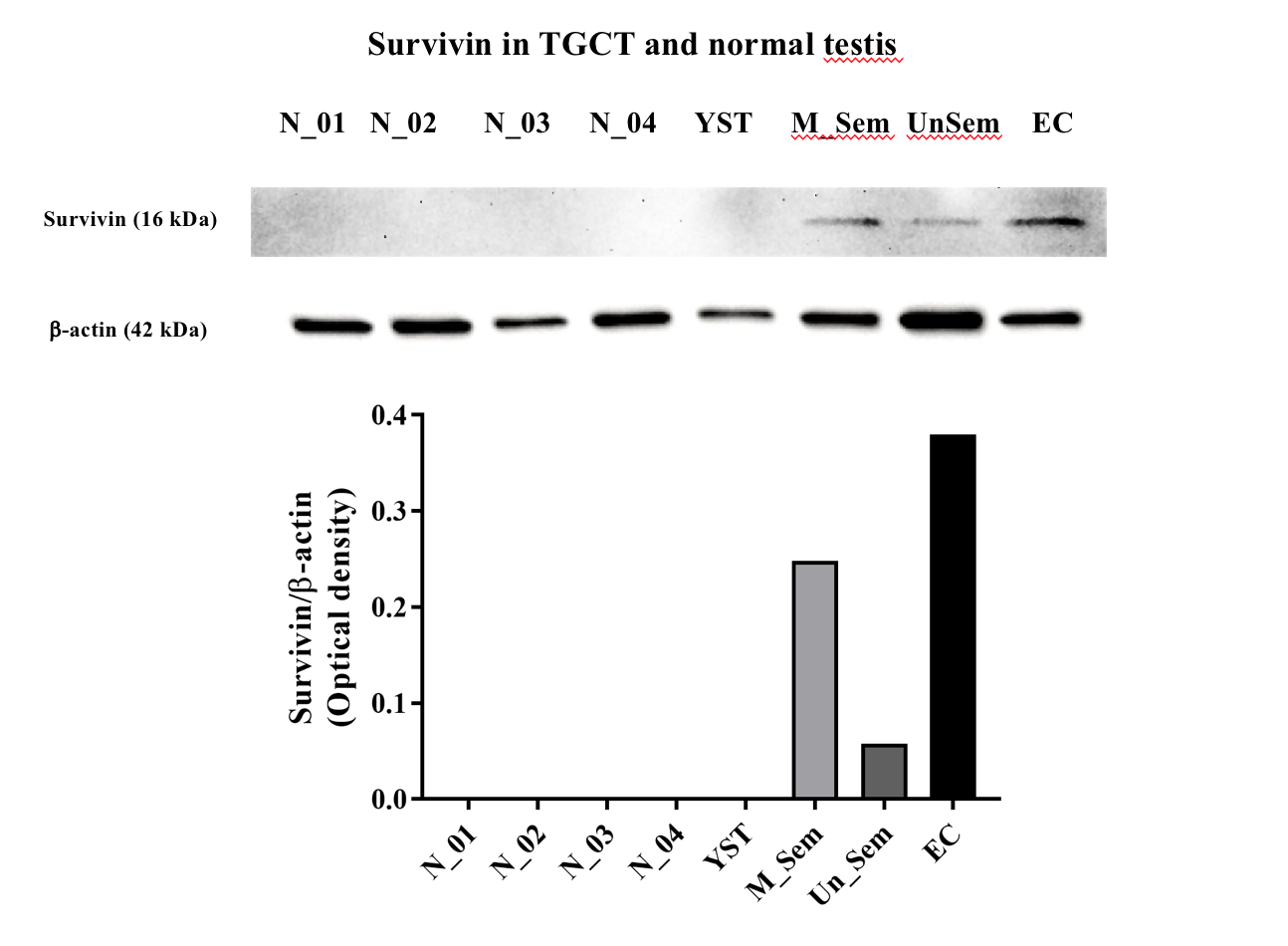


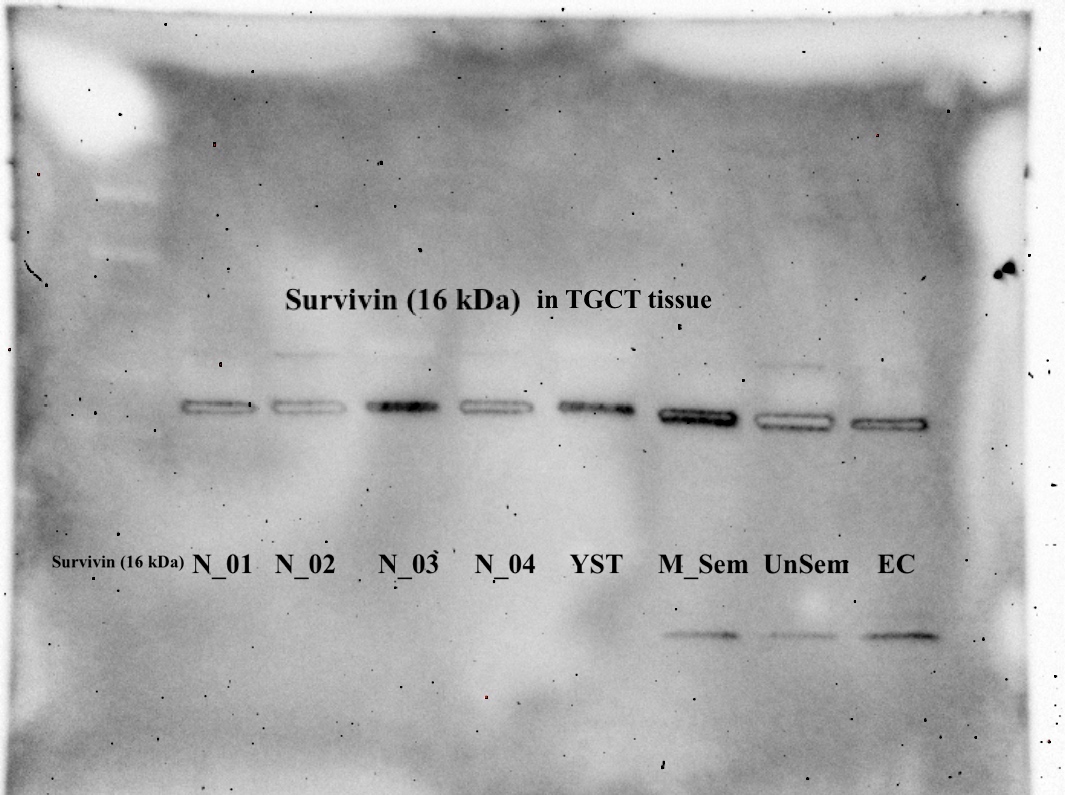

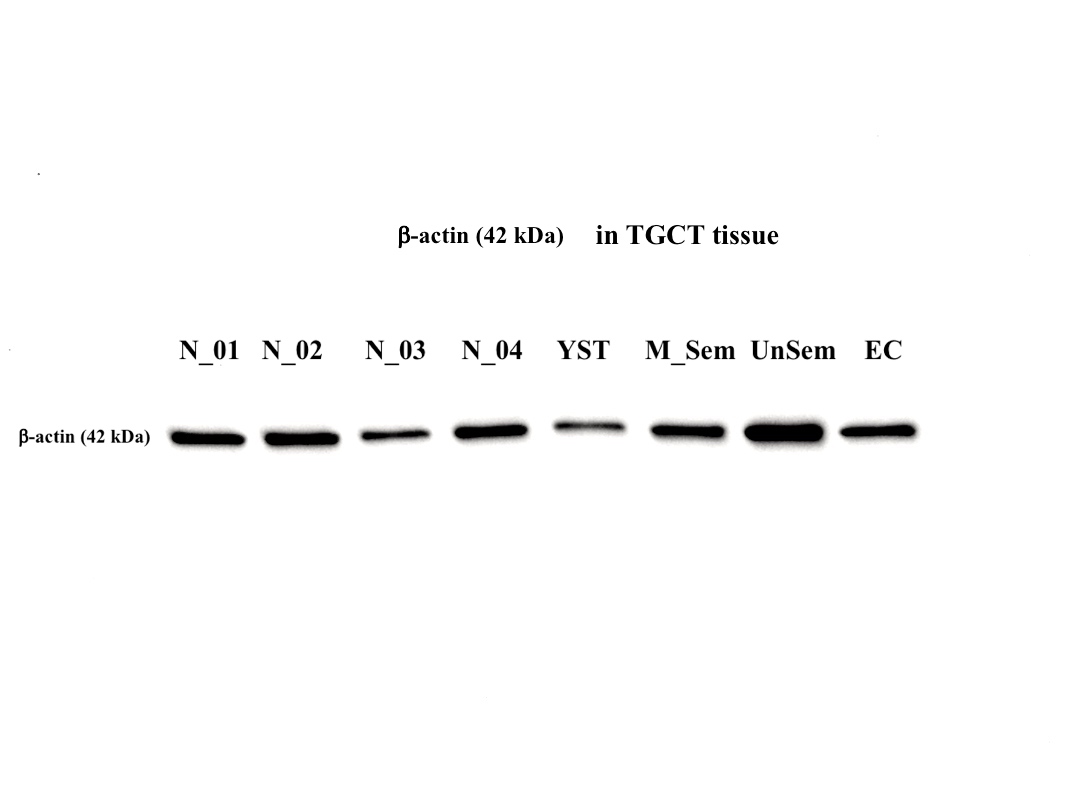


**Supplementary Figure S4**: Expression of survivin in TGCTs. The expression of survivin was measured in TGCT and normal testis samples by western blot where 15 μg of protein was loaded for detection. Survivin was detected in various amounts in the TGCT subtypes except for YST, whereas no detection of survivin was observed in normal testis. The densitometric analysis of the western blots shows that the level of survivin was highest in EC. Survivin was normalized with β-actin. N (normal); YST (yolk sac tumour); M_Sem (moderately differentiated seminoma); Un_Sem (undifferentiated seminoma); EC (embryonal carcinoma). Full length blots are also presented and red rectangle shows the cropping location.
